# Predicting Porosity in Freeze Casting with Explainable Machine Learning

**DOI:** 10.1021/acsomega.5c04133

**Published:** 2025-07-16

**Authors:** Rafael Gaspar Bessa de Oliveira, Jones Yudi, Edson Paulo da Silva, Guillermo Alvarez Bestard, Luiz Antonio Ribeiro Junior, Alysson Martins Almeida Silva

**Affiliations:** † College of Technology, Department of Mechanical Engineering, University of Brasília, Federal District, Brasília 70910-900, Brazil; ‡ Institute of Physics, University of Brasília, Federal District, Brasília 70910-900, Brazil; § Computational Materials Laboratory, LCCMat, Institute of Physics, University of Brasília, Federal District, Brasília 70910-900, Brazil

## Abstract

Freeze casting is a versatile manufacturing process for producing porous materials with tailored microstructures and properties. However, due to the complexity and variability involved, predicting porosity based on process parameters remains a challenging task. Accurate prediction is crucial for designing materials as porosity significantly impacts their applications. This study applies machine learning techniques, including CatBoost, Random Forest, and XGBoost, to predict porosity using experimental data from 252 research papers covering ceramics, polymers, and composites. The CatBoost model demonstrated the best predictive performance with an *R* ^2^ of 0.81 on the test set. Shapley Additive Explanations (SHAP) analysis revealed that solid loading had the most significant influence on predictions, with lower loading leading to increased porosity, as expected theoretically. The results highlight the potential of explainable machine learning to guide experimental design and optimize porosity in freeze casting materials.

## Graphical abstract

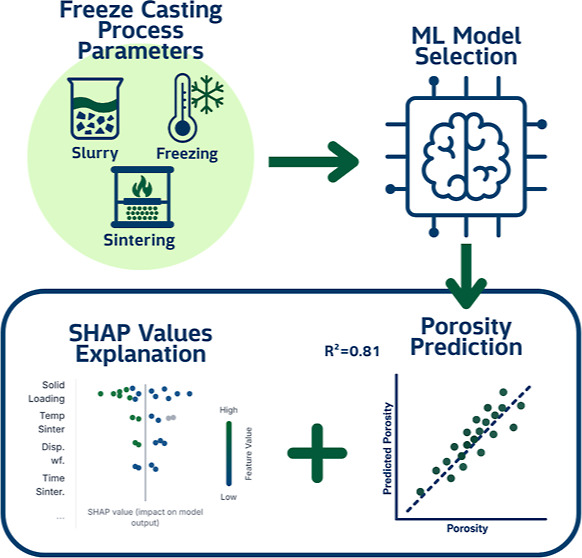

## Introduction

Porous materials are critical in advanced applications such as tissue engineering,^1^ filters,^2^ energy storage,^3^ fluid distributors,^4^ catalyst supporters,^3^ and thermal insulators.^5^ Freeze casting has emerged as a versatile and practical fabrication process to control the microstructure and porosity of such materials by manipulating key processing parameters.^6^ The technique involves preparing and freezing a ceramic slurry that combines a solid, a solvent, and optional additives. The resulting material exhibits tailored porosity and mechanical properties, making freeze casting an attractive engineering solution.

The versatility of freeze casting is due to the ability to control the material’s characteristics by selecting different parameters at each step of the fabrication process, resulting in a wide range of material properties. The properties of interest are the porosity, pore size, morphology, mechanical strength, and pore interconnectivity. The porosity, which refers to the fraction of the volume of voids over the porous material volume,^7^ is directly linked to the material’s application^8^ and thus is crucial for determining the functionality and performance of the material.^9^ Numerous studies have investigated how parameters like solid loading (volume fraction of solid materials), freezing temperature, freezing direction, and sintering influence the final material properties.^10–15^ For example, changes in the solid-to-solvent ratio affect the porosity, where higher solid loading reduces the overall porosity by limiting the amount of solvent for crystallization.^16^ Similarly, variations in freezing temperature and freezing gradient can produce hierarchical or regionally concentrated porosity.^6,11^ Advanced magnetic or electric field freezing techniques have also been explored to achieve precise control over pore morphology.^17^

Although progress has been made in freeze casting techniques and materials, predicting porosity for different parameter combinations remains challenging, given the number of parameters that can affect the microstructure.^18,19^ Moreover, predictability is crucial given that these properties, such as porosity and pore width, are directly linked to the material’s application.^8^ For example, in tissue engineering, pore size, connectivity, and orientation are crucial for bone growth into ceramic structures.^9^ Additionally, when introducing a new material or technique, the process must be repeated in the laboratory until the desired property is achieved, which is both time- and resource-consuming.

While many parameter combinations introduce significant complexity, lab-to-lab variability further complicates predictability, highlighting the need for data-driven approaches. Recent advances in artificial intelligence (AI) offer new possibilities to address these challenges by predicting material properties based on experimental data from multiple laboratories.^20,21^ Machine learning (ML) algorithms, particularly decision-tree-based models, have shown promise in capturing the complex, nonlinear relationships between input variables and target properties.^22–24^

A recent study employed neural networks to predict porosity in freeze casting materials and demonstrated promising results.^7^ However, this work aims to improve upon those results by leveraging decision-tree-based models, which are expected to outperform neural networks in structured tabular data sets.^24^ Additionally, it includes SHAP analysis to measure the contribution of each feature to the model’s prediction, serving as a tool to guide experimental decisions and improve model interpretability. Three decision-tree-based models were evaluated by using an extensive experimental database: CatBoost,^25^ Random Forest,^26^ and XGBoost.^27^ Our results show that the CatBoost model performed better with an *R* ^2^ of 0.81 on the test set. Shapley Additive Explanations (SHAP)^28^ analysis was performed to interpret the model to measure feature contribution to the model’s predictions, locally and globally, with solid loading emerging as the most influential parameter. This work demonstrates the potential of combining explainable AI with freeze casting to optimize material design and accelerate experimental workflows. Although decision-tree models and SHAP analyses have been applied in materials informatics, their use on a multimaterial freeze casting data set of this scale, with extended feature analysis, remains unprecedented. In this study, we integrate data from 252 independent publications (1865 samples) spanning ceramics, polymers, metals, and composites. We systematically evaluate model generalization across material classes and employ SHAP as a quantitative design tool to guide porosity optimization. This approach resulted in higher predictive performance than previously reported models trained on similar data sets.^7^

## Methodology

The data set used in this study was compiled from 252 research papers, encompassing 1865 samples of freeze cast materials, including ceramics, polymers, and metals.^16^ The data contained both numerical and categorical features, such as solid loading, freezing temperature, sintering temperature, dispersant weight fraction, solid name, and fluid name, all of which are critical for predicting porosity. The data preprocessing involved identifying and addressing the missing values and inconsistent entries. Features with excessive missing values were excluded to avoid introducing noise and errors into the model.

Decision-tree-based models, specifically CatBoost,^25^ Random Forest,^26^ and XGBoost,^27^ were employed due to their ability to handle complex, nonlinear relationships and mixed data types. Linear regression was also included as a baseline model for comparison. A grid search approach was used to optimize key hyperparameters, including the number of estimators, tree depth, and feature selection criteria. The hyperparameter search was guided by cross-validation, which ensured that the models were evaluated on multiple subsets of the data to prevent overfitting and assess generalization.

The models were trained using 80% of the data set, with the remaining 20% reserved for testing. The training process involved fitting the models to the selected features and minimizing prediction errors using an evaluation metric based on the Mean Absolute Error (MAE). *R* ^2^ and MAE were calculated for training and test data sets to evaluate model accuracy and generalization. The CatBoost model consistently outperformed the other models, achieving higher *R* ^2^ values and lower MAE scores, indicating its effectiveness in predicting the porosity across various experimental conditions.

To enhance the interpretability of the models, SHAP analysis was applied to quantify the contribution of individual features to the predictions. SHAP analysis revealed that solid loading was the most influential feature, significantly affecting the porosity by controlling the proportion of solvent available for crystal formation. Other important features, such as sintering temperature, dispersant weight fraction, and freezing temperature, were shown to have nonlinear impacts, reflecting the complex interplay of processing parameters in the freeze casting process. Categorical features, including material group and fluid name, were identified as context-specific modifiers, fine-tuning the model’s predictions based on the experimental setup.

### Data Selection

The accuracy and generalization of ML models depend heavily on the quality and representativeness of the training data.^29^ This study’s data set was derived from the publicly available repository *freezecasting.net*, which compiles experimental data on freeze casting ceramics, polymers, and composites.^16^ The selected data from the database encompasses 252 research papers and 1859 samples, making it one of the most comprehensive resources for studying the relationship between processing parameters and porosity. The data set’s diversity, with contributions from research groups across various countries, including China (580 samples), the United States (439 samples), and the Republic of Korea (246 samples), enables the training of a model on a wide range of experimental conditions, potentially enhancing its robustness.

A critical step in data preprocessing was filtering and cleaning the data set to address issues related to missing or incorrect values. Only 3% of the samples were nonsublimated. Since sublimation significantly reduces porosity in freeze casting, these nonsublimated samples were considered outliers and excluded to prevent potential errors in the analysis for those specific materials. Erroneous data entries were identified and removed, such as a sample with a volume fraction exceeding 1 and an unrealistic sintering temperature of over 8000 K. Although data inconsistencies are typical in large experimental data sets, relying solely on automated filtering methods could introduce errors, and some issues may remain undetected.

The data set exhibits a wide variation in the number of samples per paper, with some papers contributing fewer than 5 samples and others up to 50 (Figure 1a). However, even the papers with the highest sample counts contribute only a small portion of the total data set, indicating that no single source dominates the data. In terms of geographic distribution, the majority of samples originate from the USA and China (Figure 1b), yet the data set includes contributions from a broad range of countries. This diversity strengthens the model’s ability to generalize, as it captures data produced under varying experimental setups, researcher expertise, and laboratory conditions.

**Figure 1:**
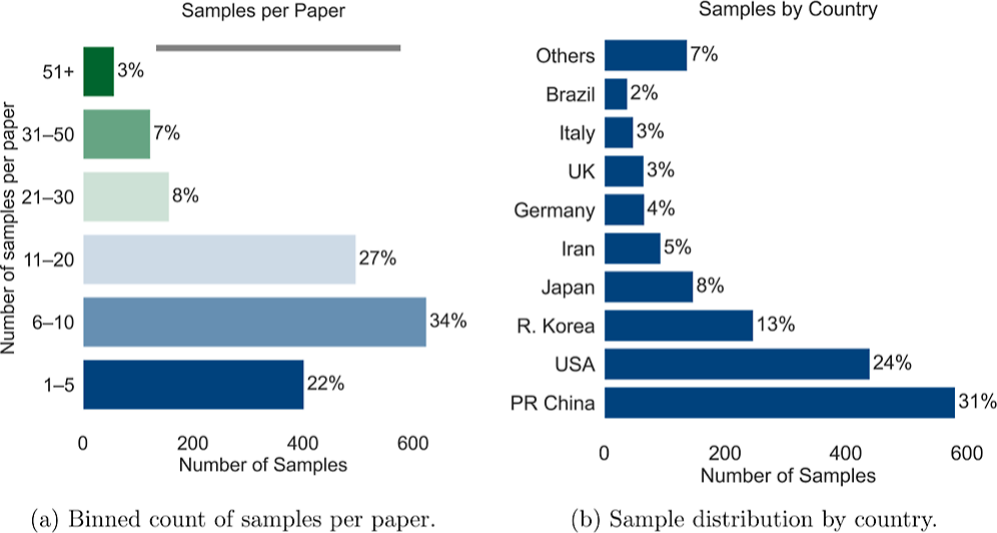
Overview of sample distribution. (a) Proportion of total samples grouped by the number of samples reported per paper (e.g., approximately 400 samples originate from papers reporting fewer than 5 samples). (b) Country distribution of samples (e.g., 31% of samples are from papers published in China).

### Feature Selection

The selected features are listed in Table 1. Porosity was chosen as the target variable due to its direct link to the material performance in several applications.^30^ The features were initially selected based on their known influence on porosity and microstructure evolution.^6^ Solid loading has a well-established negative correlation with porosity as higher solid concentrations reduce the proportion of solvent, thereby limiting pore formation during sublimation.^31^ Solid diameter, also known as particle size, affects the generation of microporosity within pore walls, which in turn affects resistance and particle packing.^32^ Freezing temperature (referred to as “Temp. cold [K]”) also plays a critical role by affecting the size and distribution of solvent crystals, with lower temperatures generally leading to finer microstructures due to faster solidification rates.^33^ Sintering temperature and time affect the extent of the densification, which reduces porosity and enhances mechanical strength by densifying the pore walls.^34^

**Table 1:** Selected and Removed Features with Respective Data Types and Percentage of Missing Values^a^

selected features	data type	missing %
solid name	categorical	0%
fluid name	categorical	0%
material group	categorical	0%
name binder	categorical	51%
porosity [%]	numerical	0%
solid loading [%]	numerical	0%
temp. sinter *f*	numerical	13%
time in sintering [hours]	numerical	23%
dispersant *wf [%]	numerical	41%
temp. cold [K]	numerical	52%
binder *wf [%]	numerical	58%

removed features	data type	missing %
dispersant name	categorical	31%
secondary solid name	categorical	72%
technique	categorical	87%
direction	categorical	87%
solid diameter [m]	numerical	20%
time in sublimation [hours]	numerical	37%
cooling rate [K/min]	numerical	84%

a A horizontal line separates selected from removed features. Weight fraction is abbreviated as *wf.

Some features were not selected despite their importance to the microstructure. The freezing technique, freezing direction, and secondary solid name were removed due to their low availability with missing percentages exceeding 70%. The cooling rate was also excluded, even though it affects crystal growth and pore morphology,^35^ due to its challenging nature in terms of control and measurement, and consequently was reported as 0 or missing in 84% of the samples.

An ablation study was conducted to assess whether removing additional features would impact the performance. Remaining features that did not impact performance when removed were also excluded from training: the dispersant name, sublimation time, and solid diameter. Other features were retained despite their moderately high missing values due to their known theoretical relevance, ease of measurement in typical laboratory settings, and support for consistency in future database extensions. This selection should be revisited as more data become available as the predictive values of features may change. It is essential to note that many features are relevant to the process, but due to data limitations, they could not be utilized; however, this may change as the data set expands.

### Exploratory Analysis

An exploratory analysis of the selected data set is crucial for identifying underlying relationships between features, potential biases, and limitations. The analysis also helps ensure that the data set is aligned with theoretical expectations. Additionally, the models handle missing values in the predictions of the ML models.

Although external out-of-sample testing would further confirm the model robustness, the current lack of uniformly reported process porosity data outside our compiled database limits immediate validation. Our comprehensive cross-validation strategy and stable performance across material groups nonetheless support the model’s generalizability. Future efforts will focus on obtaining and standardizing external data sets for formal out-of-sample evaluation.

### Missing Values

The percentage of missing data for each selected feature is displayed in Table 1. Approximately 13% to 58% of the samples had missing values for features such as sintering temperature, freezing time, and binder content. Missing values in key features can introduce bias and limit the model’s learning capacity as it has less information to work with. Additionally, models handle missing values in different ways. CatBoost and XGBoost can handle missing values natively, but other approaches can also be used. In CatBoost, numerical missing values are processed as the minimum value but are guaranteed a split that separates them in the decision tree.^36^ In XGBoost, branch directions for missing values are learned during training,^27^ whereas Random Forest requires an imputation preprocessing step to handle empty data.

### Categorical Features Analysis

The selected categorical features were the material group, solid name, and fluid name (i.e., solvent). Different solids and fluids produce varying pore microstructures due to differences in particle size, surface chemistry, binders, and their interactions.^6,13^ The data set exhibits a significant class imbalance, with ceramics comprising over 85% of the material group samples and water-based solvents, accounting for more than 64% of the fluid types. This disproportion of representation can introduce a bias as there are fewer samples for a model to learn patterns from less frequent categories. Additionally, due to limited data, the model may overfit by memorizing specific patterns associated with rare categories, resulting in a biased behavior that does not generalize to real-world scenarios.

Material groups exhibit distinct characteristics. Polymeric materials tend to have high porosity values with a lower variance. In contrast, Metal/Ceramic shows the lowest mean porosity value but with outliers that reach high porosity ranges.

Despite the importance of the selected categorical features to the resulting microstructure, Figure 2 and Table 2 show similar variances and means within the most frequent categories of solids and fluids. Among the solids used, there is little difference between the porosity distributions. This category has the most balanced representation; the most frequent group is hydroxyapatite (Al_2_O_3_), which accounts for 25% of the total samples and exhibits a wide range of porosity values.

**Figure 2:**
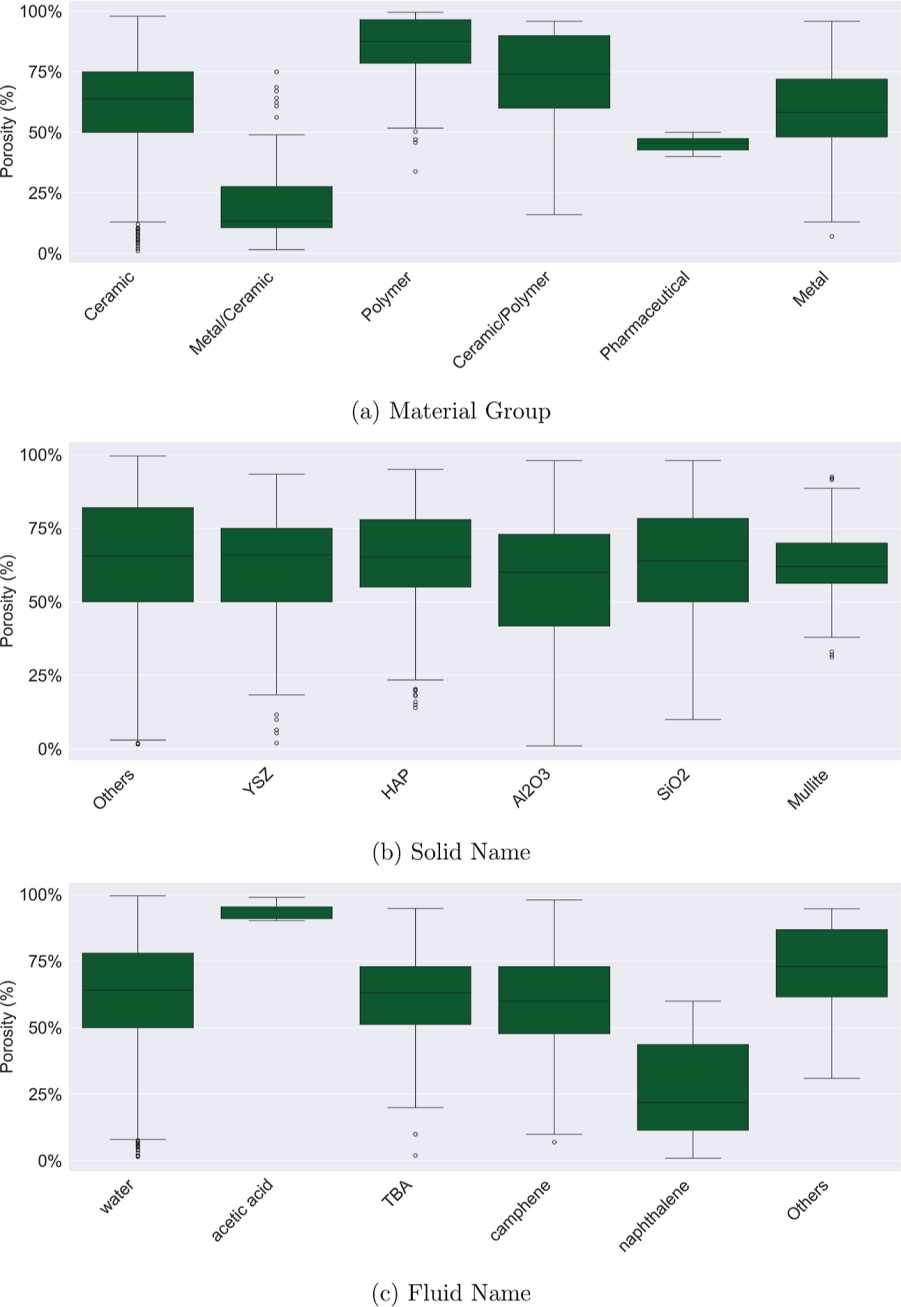
Boxplot of porosity values by categorical features.

**Table 2:** Summary of Categorical Features: Sample Count, Percentage of Total Samples, Mean Porosity Percentage, and Standard Deviation of Porosity Percentage^a^

feature	category	count	total %	porosity	
				mean (%)	STD (%)
group	ceramic	1587	85	61	19
	polymer	91	5	85	15
	metal	90	5	59	21
	ceramic/polymer	45	2	72	21
	metal/ceramic	40	2	24	21
	pharmaceutical	6	0	45	4
solid name	others	677	36	64	21
	Al_2_O_3_	456	25	55	23
	HAP	353	19	64	17
	YSZ	180	10	61	18
	SiO_2_	110	6	64	18
	mullite	83	4	63	12
fluid name	water	1184	64	62	21
	TBA	371	20	61	16
	camphene	224	12	58	20
	acetic acid	38	2	93	3
	others	30	2	70	18
	naphthalene	12	1	28	22

a Results are presented for the top categories, with all other categories grouped as “others”. “STD” abbreviates standard deviation of the porosity percentage.

The fluid used is known to have a minimal impact on porosity.^37^ Except for acetic acid and naphthalene, the categories in Figure 2 have similar means and ranges for the porosity values. While the contribution of this feature to porosity is expected to be low, it is relatively simple to include. It may help fine-tune the model for specific cases when combined with other features. In the data set, water has the highest representation, accounting for over 60% of the samples, followed by 20% of *tert*-butyl alcohol (TBA). In contrast, acetic acid has a narrow range of porosity values, which may be a result of its limited sample size in the data set.

### Numerical Features Analysis

In order to analyze the numerical features, we used a Pearson’s correlation matrix as shown in Figure 3. Solid loading exhibits the highest negative correlation with porosity (−58.18%), consistent with existing freeze casting materials research,^31^ showing that the values in the selected database are in agreement with the literature, and reaffirms solid loading as a fundamental feature for porosity prediction. Higher solid loading reduces the solvent content in the slurry, thereby limiting the formation of solvent crystals during freezing and leading to fewer voids upon sublimation.^6^ Other numerical features show weaker correlations with porosity despite their previously mentioned influence on the microstructure. This may suggest that their impact on porosity is minimal or that it varies across materials. It is both complex and nonlinear, an aspect not fully captured by Pearson’s correlation analysis.

**Figure 3:**
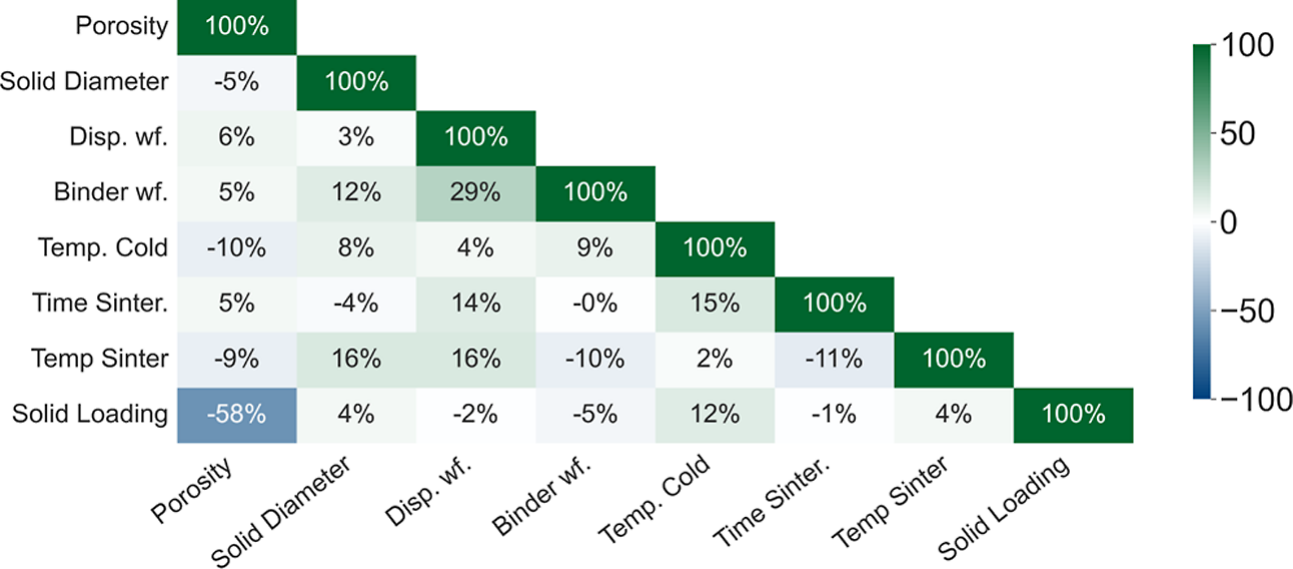
Correlation matrix for numerical features.

### Performance Measures

Two statistical measures were used to compare a model’s performance. The coefficient of determination, *R* ^2^, measures how well a model’s prediction matches the true porosity values. A value close to 1 indicates a perfect prediction, and 0 means a poor prediction that is no better than using just the mean of the data. The second metric used was the mean average error (MAE), where values closer to zero indicate better performance.

### Model Selection

Gradient-boosted decision trees are widely recognized for their superior performance in tabular data modeling.^24^ For the regression task of predicting material porosity, we selected well-established decision-tree-based models known for their effectiveness: CatBoost,^25^ XGBoost (XGB),^27^ and Random Forest.^26^

### Training and Hyperparameter Optimization

The performance of ML models heavily relies on carefully selecting hyperparameters to strike a balance between predictive accuracy and model complexity. Hyperparameters, which govern the internal configuration of models, influence the model’s ability to learn patterns from training data and mitigate both overfitting and underfitting. This study employed a grid-search approach to optimize the key hyperparameters of the three decision-tree-based models: CatBoost, XGBoost (XGB), and Random Forest, as well as a Linear Regression (Linear Reg.) model, served as a baseline.

The training process was implemented using the scikit-learn framework. The data were split into 80% for training and 20% for testing, deviating from the conventional 70%–30% split to compensate for the relatively small sample size. This adjustment enhances the model by incorporating a broader range of materials and conditions during training, thereby improving its generalization ability.

The model pipeline consisted of a preprocessing step and the model itself. The preprocessing step consisted of a combination of three possible strategies: no preprocessing, one-hot encoding, and one-hot encoding with mean imputation. The preprocessing strategies specific to each model are summarized in Table 4. Many machine learning models require one-hot encoding to convert categorical features from text to a numeric format, whereas CatBoost has a native way to handle this through target encoding. One-hot encoding represents each category of a feature as a unique binary vector. For example, the single feature material group can be transformed into multiple binary features, such as “material_group_ceramic”, “material_group_polymer”, and “material_group_infrequent”. These new features will have values of 1 for samples that belong to that category and 0 for those that do not. One-hot encoding was applied to categorical features with a “min_frequency” threshold set to 4%. This means that categories with lower representations were grouped as “infrequent”. This threshold was chosen to ensure that Ceramic, Metallic, and Polymer materials remained distinct categories, consequently “Ceramic/Polymer”, “Metal/Ceramic”, and “Pharmaceutical” were considered infrequent. Additionally, this approach reduces the bias of the model, learning specific patterns for rare categories.

**Table 3:** Grid-Search Parameters

model	parameter	hyperparameters	chosen value
CatBoost	*n_estimators*	50–500	250
	*depth*	1–10	8
	*eval* *_metric*	mae, mape, rmse	mae
XGB	*n_estimators*	50–500	100
	*max_depth*	2–10	5
Random Forest	*n_estimators*	50–200	100
	*max_features*	log_2_, sqrt, none	log_2_
	*max_depth*	10–40	20

**Table 4:** Preprocessing Strategies

model	preprocessing strategy
CatBoost	native
	one-hot encoding
	one-hot encoding + mean imputing
XGB	one-hot encoding
	one-hot encoding + mean imputing
Random Forest	one-hot encoding + mean imputing
Linear Reg.	one-hot encoding + mean imputing

A grid search approach was used for training to find the best candidate values for the most critical hyperparameters. The *R* ^2^ was used as the score metric for model selection. For decision-tree models, the depth of the trees and the number of estimators work similarly to the control of layers and the number of neurons in neural networks, acting as regularization mechanisms to balance underfitting and overfitting. Deeper trees and a larger number of estimators enable the model to capture more nuanced patterns in the data; however, this also increases the risk of overfitting, when the model begins to capture noise or patterns that are unique to a particular study. The ranges and best performing hyperparameters selected for each model are listed in Table 3.

Cross-validation was implemented with 5 folds, which splits the training data set into 5 nonoverlapping data sets. Each data set is used as a validation set once, while the other 4 data sets are used to fit a model. Cross-validation mitigates the risk of obtaining a “lucky” split with the random number generator. It also enables checking model stability and whether performance has high deviation across different folds and provides information on how well the model performs on unseen data throughout training without sacrificing data for a validation set. The model’s performance metrics are then calculated using the average of the models trained on each fold.

Categories with <4% frequency were merged into a single “infrequent” label to prevent overfitting on sparse data. Although this reduces resolution for uncommon materials, the model still captures their collective effect via the “infrequent” feature. As more data become available, these categories can be further subdivided to enhance applicability to specific rare material systems.

Categorical variables were processed as follows: CatBoost was tested with both its native categorical encoding and with one-hot encoding (4% min-frequency threshold), with the latter used for all final results; XGBoost and Random Forest do not natively support categorical features and therefore received one-hot encoded inputs after imputation (for Random Forest). This uniform encoding strategy facilitates a fair comparison across models.

### Results and Discussion

#### Overall Performances

The *R* ^2^ was measured using the model’s predicted porosity and the true value for that sample. Table 5 shows that all machine learning models outperformed the linear regression performed using solid loading. The best-performing model, as measured by the *R* ^2^ metric, was CatBoost. The imputation step showed no relevant impact on performance, regardless of the model in which it was applied. The CatBoost model without imputation was selected for usage and further analysis, as it is clearer to evaluate the impact of missing features without the imputation step. We selected the CatBoost (one-hot) model for further analysis as it had the highest performance and was easier to interpret without imputation. The obtained performance was a mean *R* ^2^ of 0.92 with a standard deviation of 0.02 when evaluated in the 5-fold range used in training.

**Table 5:** Model Performances on Train and Test Sets^a^

model	train	test	
	*R* ^2^	*R* ^2^	MAE
CatBoost (one-hot + impute)	0.92	0.81	0.06
**CatBoost** **(one-hot)**	**0.92**	**0.81**	**0.06**
RF (one-hot + impute)	0.92	0.80	0.06
XGB (one-hot)	0.93	0.78	0.06
CatBoost (native encoding)	0.88	0.77	0.07
XGB (one-hot + impute)	0.93	0.77	0.06
Linear Reg.	0.43	0.35	0.13
Linear Reg. (SL)	0.35	0.28	0.14

a SL*: model trained using only the solid loading feature.

As shown in Table 6, performance varied with *R* ^2^ values ranging from 0.53 to 0.98 within the most frequent groups. The average prediction error was near zero, indicating no significant bias toward overestimating or underestimating porosity, as illustrated in Figure 4. The CatBoost model was also trained using the native encoding of categorical features. However, the performance decreased to *R* ^2^ = 0.77 in the test. Hence, the one-hot encoding approach with the *encode_min_frequency* parameter for CatBoost still provided a better overall performance.

**Table 6:** CatBoost (One-Hot) Prediction Performance *R* ^2^ across the Top 5 Most Frequent Material Categories

category	train samples	test samples	*R* ^2^
Material Group			
ceramic	1276	311	0.79
polymer	77	14	0.77
metal	66	24	0.85
ceramic/polymer	36	9	0.93
metal/ceramic	27	13	0.67
Solid Name			
Al_2_O_3_	370	86	0.88
HAP	273	80	0.65
YSZ	146	34	0.88
SiO_2_	87	23	0.78
mullite	70	13	0.74
Fluid Name			
water	938	246	0.81
TBA	302	69	0.78
camphene	184	40	0.70
acetic acid	31	7	0.53
naphthalene	10	2	0.98

**Figure 4:**
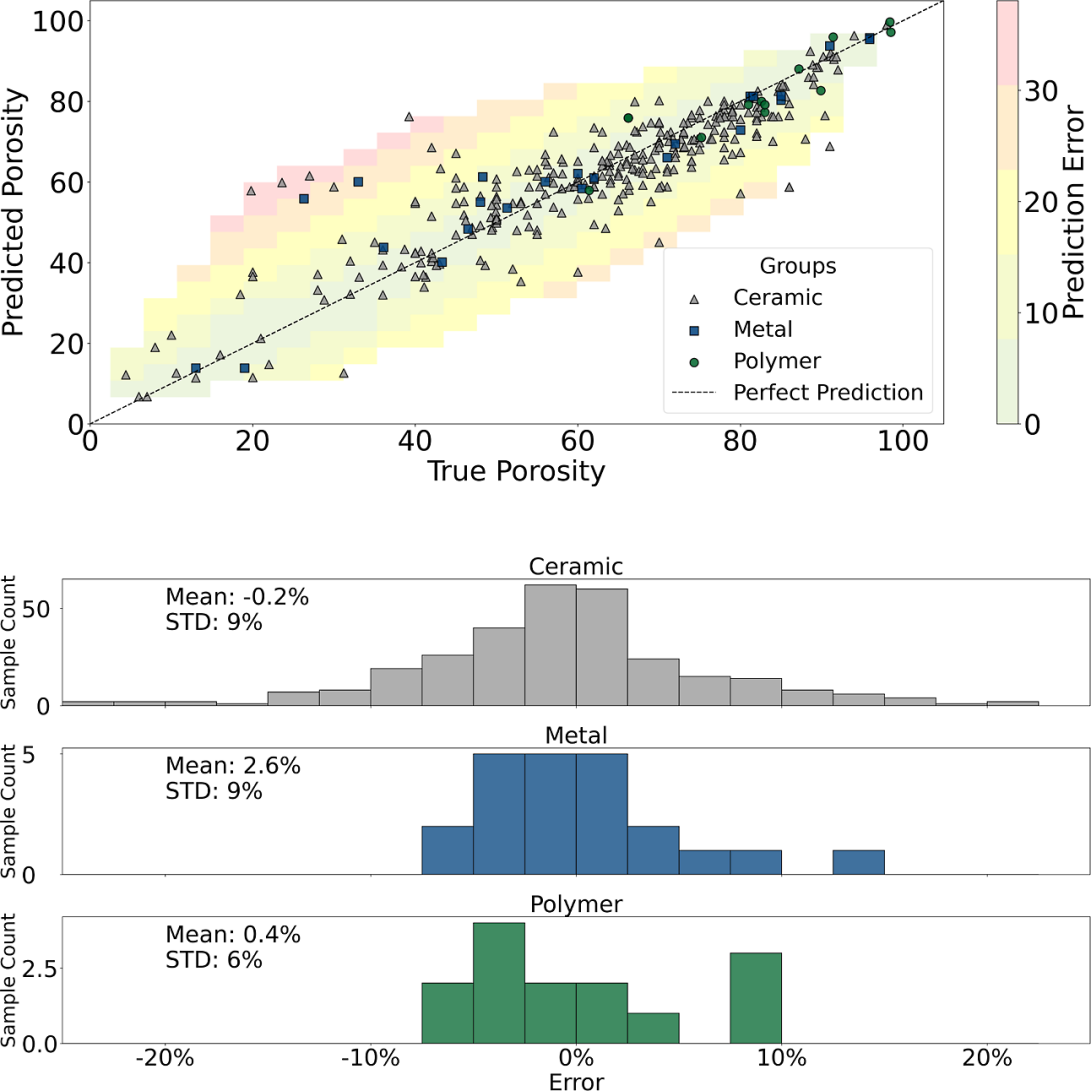
CatBoost (one-hot) performance and error distribution of the predicted porosity in the test by material group. Each material group has the mean and standard deviation (STD) of the error reported.

Although ceramics dominate the data set, the CatBoost model also maintained competitive *R* ^2^ values for polymers and metals. However, a slightly higher variance in prediction error was observed for underrepresented categories, such as polymers and composites, likely due to the smaller number of training samples. This highlights the importance of expanding 21 the data set in future work to improve performance consistency across material types.

#### Performance by Material Group Categories

CatBoost (one-hot) achieved high *R* ^2^ values for ceramics (0.79), polymers (0.77), and metals (0.85), despite class imbalance, suggesting strong generalization (Table 6). At the individual material level, it maintained a high accuracy for Al_2_O_3_ and YSZ (*R* ^2^ = 0.88). However, the model underperformed in some instances, such as HAP (*R* ^2^ = 0.65) and, particularly, for the fluid acetic acid (*R* ^2^ = 0.53). Despite the latter exhibiting a narrow porosity range (2), the model likely failed to capture its trend due to limited sample availability. Moreover, *R* ^2^ values did not correlate with the number of training samples, indicating robustness to sample imbalance.

Despite the promising results, limitations were identified, including the data set’s lack of representation for specific materials and experimental conditions. Additionally, emerging new techniques and materials may challenge the performance of the trained model. Expanding the data set to include more samples from underrepresented categories and additional features could address these issues and enhance model accuracy.

#### Performance by Sample’s Year of Publication

Model performance remained consistent across publication years, with no clear trend observed (Figure 5), except a drop in performance in 2010. The yearly proportion of newer materials stayed relatively stable, while the distribution of recent years saw a higher share of materials outside the top 10 most frequent. In the later years of the data set, the number of samples per material indicates a decline in HAP samples and an increase in other materials (materials not in the topmost frequent) and Al_2_O_3_.

**Figure 5:**
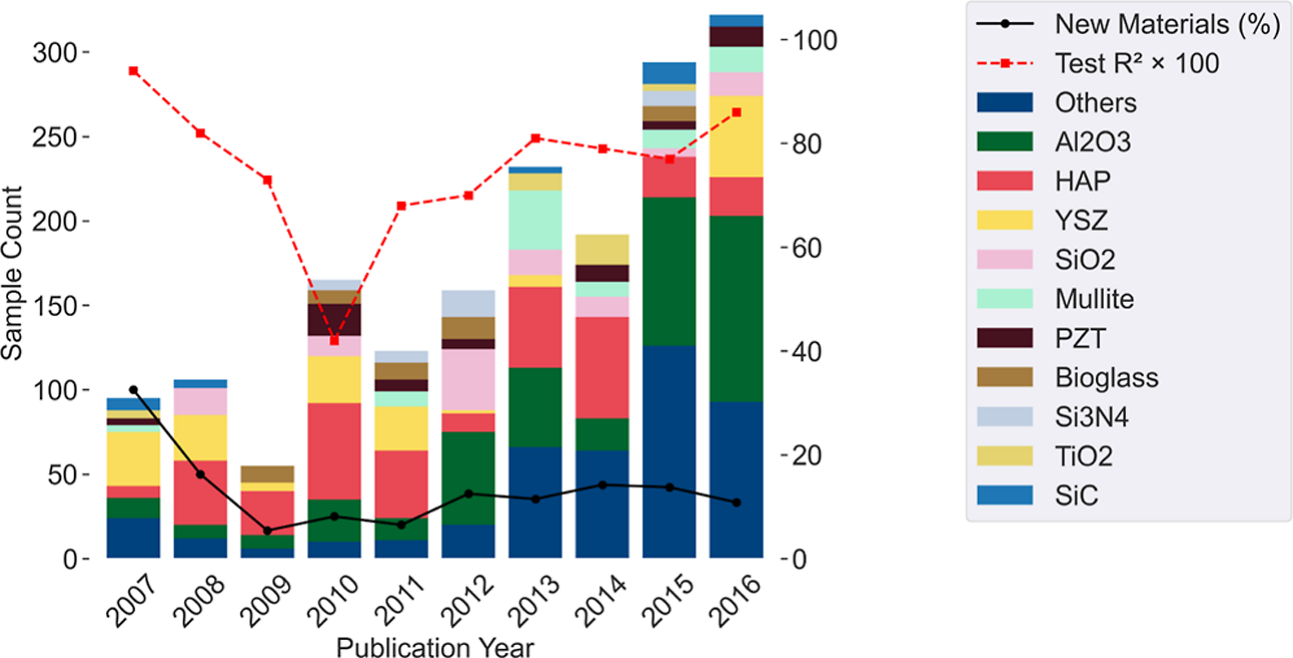
Analysis of performance by publication year. Bars represent the count of samples of solids, and lines show the percentage of new materials in that year as well as the obtained *R* ^2^ from the CatBoost prediction. Materials not included in the top 10 most frequent were grouped as “others”.

#### Performance by Material’s Training Frequency

We analyzed the model performance as a function of the solid material frequency in the training set. Fewer than 1% of the test samples came from materials not seen during training; this group showed poor predictive performance with nonsignificant *R* ^2^ values. In contrast, over 60% of the test samples were associated with materials that had more than 50 training samples.

As shown in Figure 6, the performance stabilizes when a material has more than 10 samples in the training set. The XGBoost model with imputation maintained consistent performance across both low and high sample counts, indicating strong generalization even with limited data, whereas for CatBoost, the native encoding showed inferior performance in most frequency bins.

**Figure 6:**
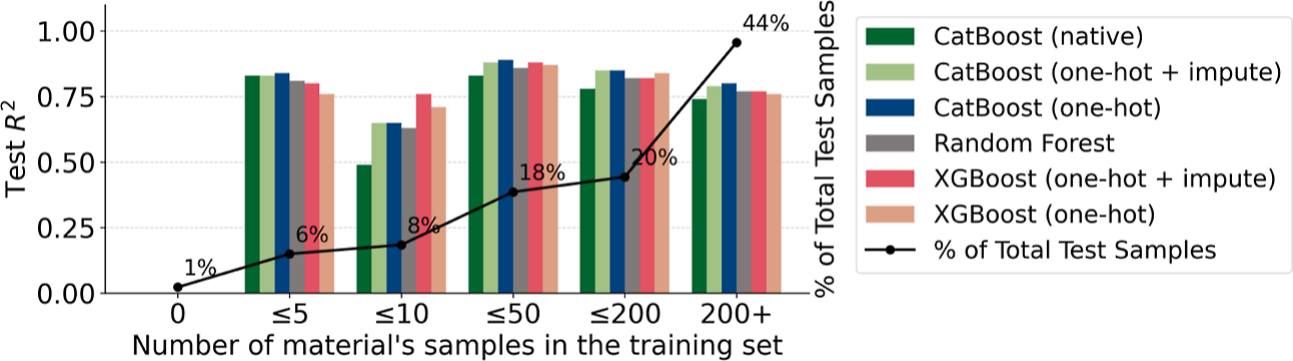
Test *R* ^2^ performance by frequency of the material in the training set.

### Model Interpretation

Shapley Additive Explanation is a tool that measures feature importance (the SHAP value) as the contribution of a feature to the model’s prediction. To quantify its contribution, the feature is withheld, and the difference in prediction is computed and averaged over all possible subsets of features.^38^ Positive SHAP values mean a positive contribution to the porosity, whereas negative values mean a negative contribution. Figure 7 is a SHAP summary plot, and the *x*-axis represents the importance value. The color gradient refers to the feature’s value. A dot represents each sample. One-hot-encoded features are represented by their respective feature and category, “Fluid Name_water”.

**Figure 7:**
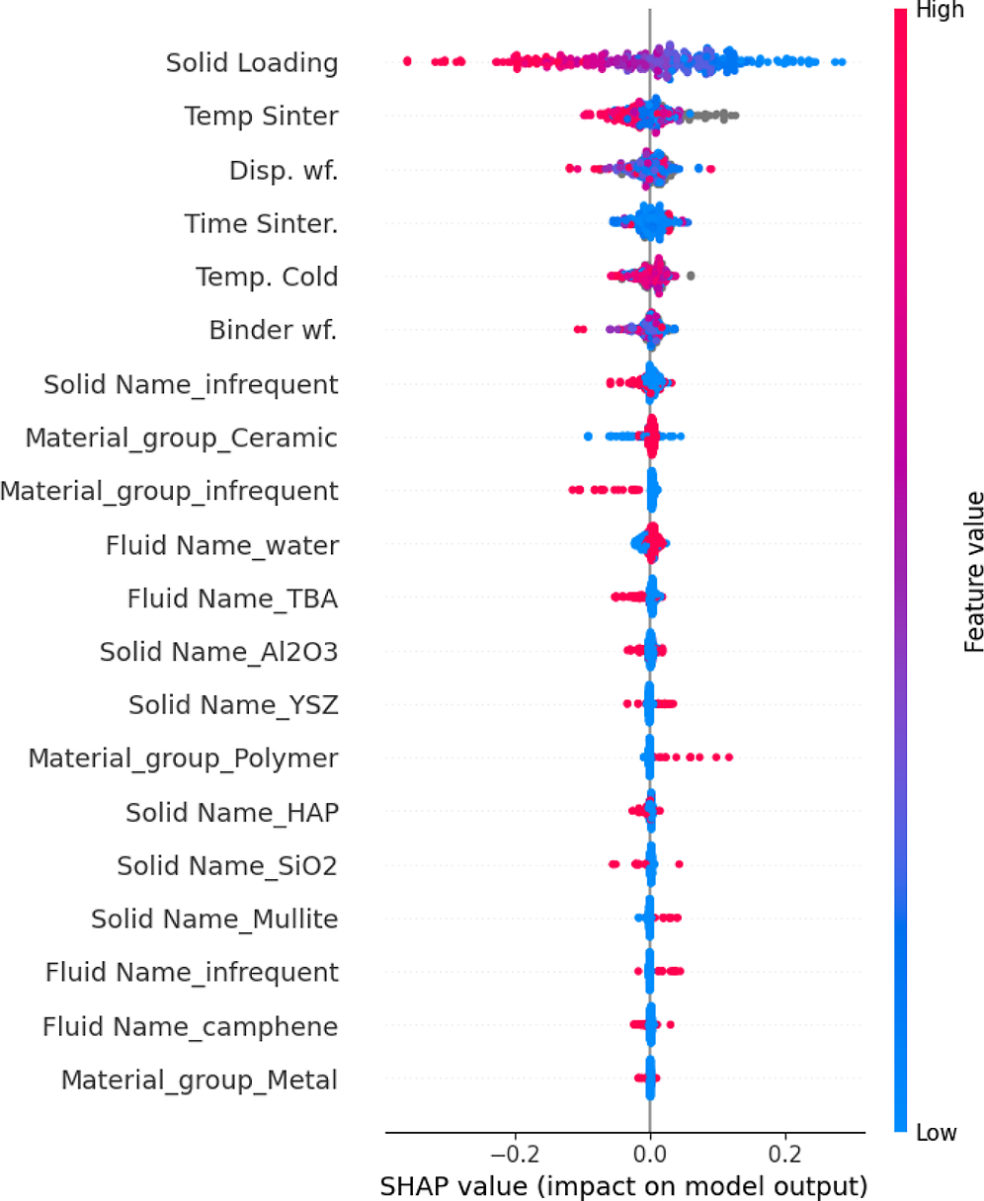
SHAP values for the top 20 most important features based on their contribution to the model’s predictions. Each feature is colored according to its value, with red indicating high values and blue indicating low values. The SHAP value, represented on the *x*-axis, shows the feature’s impact on porosity: values to the right indicate a positive contribution, while values to the left indicate a negative contribution.

The plot reveals a negative relationship between Solid Loading and SHAP values. As Solid Loading increases (from left to right on the *x*-axis), the SHAP value decreases (lower values on the *y*-axis). This indicates that higher Solid Loading tends to reduce the predicted porosity, as reflected in the SHAP values. Specifically, when Solid Loading is low, the SHAP value is more positive, suggesting that a lower Solid Loading increases the predicted porosity. As Solid Loading increases, the impact on the prediction becomes more negative, resulting in a lower expected porosity. More clearly shown in Figure 8a, a higher variance is observed as the solid loading increases.

**Figure 8:**
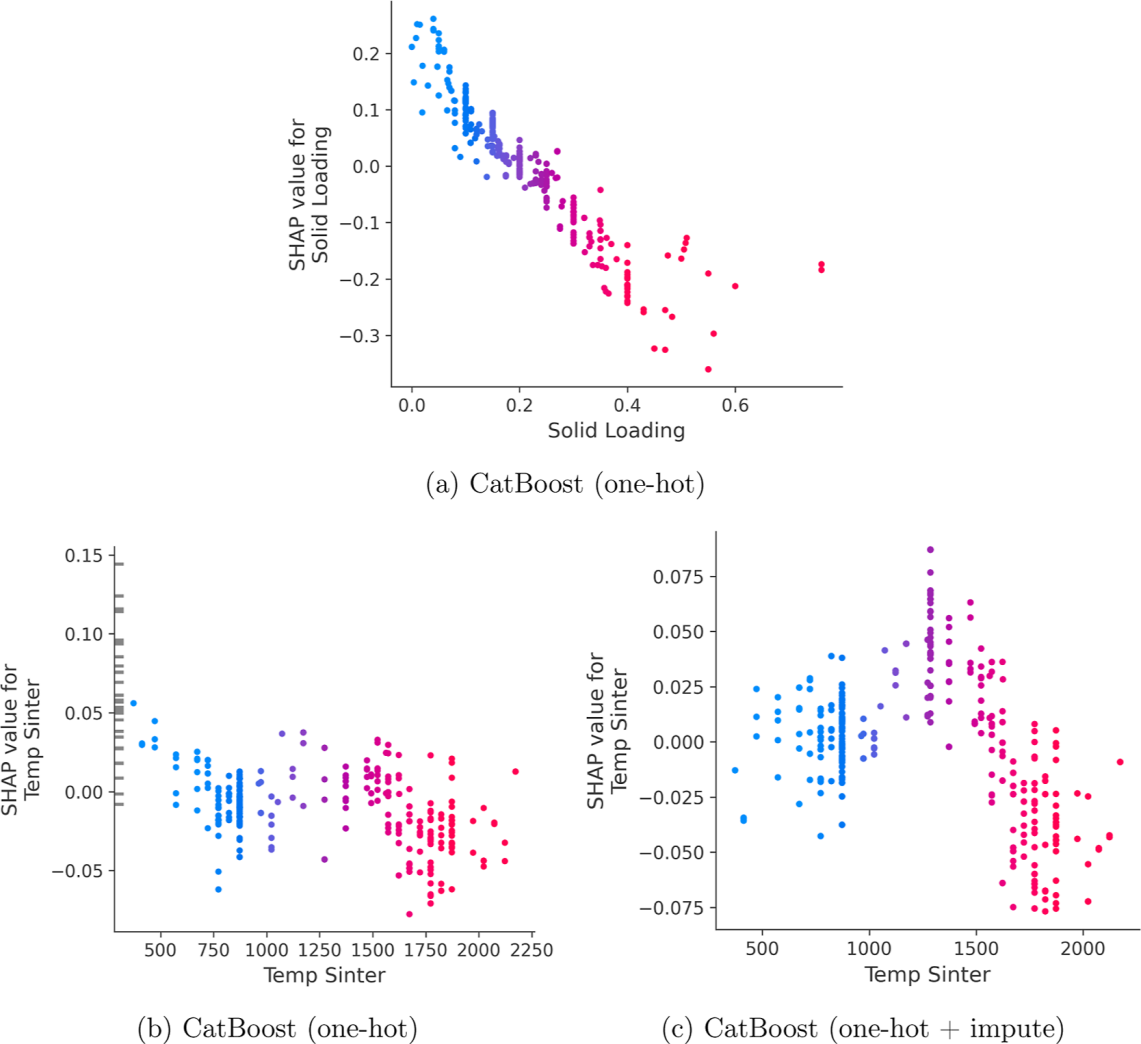
SHAP dependence plots for CatBoost models. (a) Without imputation: solid loading and sinterization temperature. (b) With mean imputation: sinterization temperature. Color represents feature value (red = high, blue = low, gray = missing).

Other features, such as “Solid Name”, exhibit a more complex relationship with porosity, contributing both positively and negatively depending on the instance. Water shows a slight positive contribution toward porosity. In addition, infrequent material groups, the solvent TBA, and higher sinterization temperatures contribute to lower porosity values. A few samples with missing values from the sinterization temperature in Figure 9a, shown in gray, made positive contributions to porosity, which indicates a clear bias learned by the model, as it is not expected that the data is missing for a reason. The SHAP values from CatBoost with imputing also showed the same behavior with the mean imputed values displaying high importance (Figure 8c). SHAP values can help researchers to assess when predictions can be trusted, uncover learned biases, and compare model behaviors across conditions.

**Figure 9:**
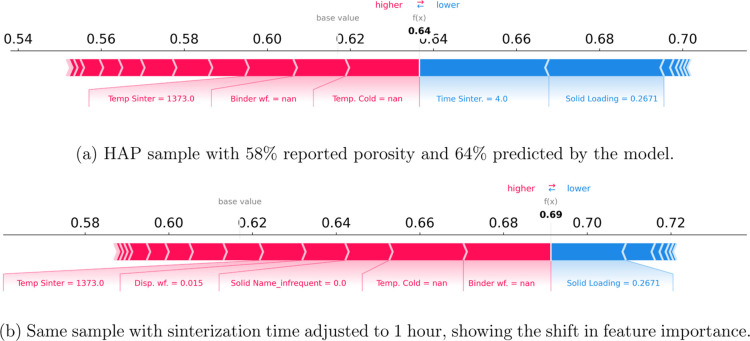
SHAP value force plots for a HAP sample using the CatBoost (one-hot) model: (a) prediction on original data, and (b) after adjusting sinterization time.

SHAP values help identify which process parameters most influence the predicted porosity, guiding researchers on what to adjust during the experiment planning. Figure 9b shows an example for an HAP sample, where Time Sintering and Solid Loading were the main factors reducing porosity. With this insight, a researcher may decide to reduce the sinter time to 1 h, leading the model to predict a porosity of 69%. This is possible because SHAP provides both local (sample-specific) and global (data set-wide) explanations, unlike traditional feature importance methods that only offer aggregated insights. This type of analysis enables more informed “what-if” experimentation and a better understanding of how each parameter impacts the outcome.

## Conclusions

In summary, this study successfully applied decision-tree-based models to predict porosity in freeze cast materials. The CatBoost model outperformed the other models. Leveraging an extensive data set encompassing ceramics, polymers, metals, and composites, the models were trained to capture the key processing parameters influencing porosity in freeze casting. By unifying a large, diverse freeze casting data set and deploying an explainable ML pipeline, this work advances beyond prior prediction methods. It offers an open framework for predicting freeze casting material porosity and providing actionable insights into process and structure relationships, laying the groundwork for community-driven tuning of freeze cast materials.

The decision tree models achieved a *R* ^2^ of 0.81 in the test set, indicating strong predictive performance. This result demonstrates an improvement in using decision trees in conjunction with additional data, compared to the results obtained in ref 7. This study also analyzed model performance across material groups, sample frequency, and publication years, highlighting strengths and limitations, particularly in cases involving novel materials, such as the use of acetic acid as a solvent. To improve performance in these underrepresented scenarios, it is crucial to expand the data set with new experimental data continually. This not only allows the model to learn patterns from emerging materials but also enables the inclusion of relevant features that were previously excluded due to data sparsity.

The same modeling strategy developed for porosity prediction can be effective and readily adapted to predict other properties such as pore diameter and mechanical strength since the input features are shared and the data available in the database. Furthermore, SHAP analysis can also be used to compare feature importance across these predictive tasks and tune specific values.

In this study, SHAP analysis further revealed valuable insights into the contributions of individual features. It confirmed that the model captured the expected relationship between porosity and solid loading as well as complex relationships with other features. Additionally, it highlighted the importance of various features in the model’s predictions, offering a tool to identify potential biases and refine porosity tuning. By understanding which features have the most significant impact, this analysis can guide laboratory adjustments for optimizing porosity in future material formulations.

Overall, the successful application of machine learning models for porosity prediction in this study represents a significant advancement in the efficiency and accuracy of the freeze casting process optimization. This approach enhances efficiency and accuracy, streamlines material design, and accelerates experimental workflows. This opens up new avenues for research and development, offering the potential to tailor porous materials with precision. Future work should focus on expanding the chemical diversity of the training data, particularly under-represented materials, techniques, and freezing fluids such as acetic acid, to further enhance the model’s predictive reach across emerging freeze casting materials.

## Data Availability

For further information, the data set and code utilized in this study are publicly accessible at: https://github.com/Bessagg/FreezeCastingPredict.

## References

[1] Hollister S. J. (2005). Porous
scaffold design for tissue engineering. Nat.
Mater..

[2] Song Y., Phipps J., Zhu C., Ma S. (2023). Porous materials for
water purification. Angew. Chem..

[3] Sun M.-H., Huang S.-Z., Chen L.-H., Li Y., Yang X.-Y., Yuan Z.-Y., Su B.-L. (2016). Applications of
hierarchically structured
porous materials from energy storage and conversion, catalysis, photocatalysis,
adsorption, separation, and sensing to biomedicine. Chem. Soc. Rev..

[4] Senn S., Poulikakos D. (2004). Polymer electrolyte
fuel cells with porous materials
as fluid distributors and comparisons with traditional channeled systems. J. Heat Transfer.

[5] Hu F., Wu S., Sun Y. (2019). Hollow-structured
materials for thermal insulation. Adv. Mater..

[6] Li W., Lu K., Walz J. (2012). Freeze casting of porous materials: review of critical
factors in microstructure evolution. Int. Mater.
Rev..

[7] Liu Y., Zhai W., Zeng K. (2020). Study of the
Freeze Casting Process
by Artificial Neural Networks. ACS Appl. Mater.
Interfaces.

[8] Ishizaki, K. ; Komarneni, S. ; Nanko, M. Porous Materials: Process Technology and Applications; Kluwer Academic Publishers, 2013.

[9] Deville S., Saiz E., Tomsia A. P. (2006). Freeze
casting of hydroxyapatite
scaffolds for bone tissue engineering. Biomaterials.

[10] Li W., Lu K., Walz J. Y. (2013). Effects
of Solids Loading on Sintering and Properties
of Freeze-Cast Kaolinite–Silica Porous Composites. J. Am. Ceram. Soc..

[11] Deville S. (2008). Freeze-casting
of porous ceramics: a review of current achievements and issues. Adv. Eng. Mater..

[12] Qian L., Zhang H. (2011). Controlled freezing
and freeze drying: a versatile route for porous
and micro-/nano-structured materials. J. Chem.
Technol. Biotechnol..

[13] Deville S. (2010). Freeze-casting
of porous biomaterials: structure, properties and opportunities. Materials.

[14] Ternero F., Rosa L. G., Urban P., Montes J. M., Cuevas F. G. (2021). Influence
of the total porosity on the properties of sintered materials—A
review. Metals.

[15] Fu Q., Rahaman M. N., Dogan F., Bal B. S. (2008). Freeze casting of
porous hydroxyapatite scaffolds. II. Sintering, microstructure, and
mechanical behavior. J. Biomed. Mater. Res.
B Appl. Biomater..

[16] Scotti K. L., Dunand D. C. (2018). Freeze casting—A
review of processing, microstructure
and properties via the open data repository, FreezeCasting. net. Prog. Mater. Sci..

[17] Wang A., Ma Y., Zhao D. (2024). Pore engineering
of Porous Materials: Effects and Applications. ACS Nano.

[18] Jablonka K. M., Ongari D., Moosavi S. M., Smit B. (2020). Big-data science in
porous materials: materials genomics and machine learning. Chem. Rev..

[19] Li Z., Yoon J., Zhang R., Rajabipour F., Srubar W. V., Dabo I., Radlinska A. (2022). Machine learning
in concrete science: applications, challenges, and best practices. npj Comput. Mater..

[20] Guo K., Yang Z., Yu C.-H., Buehler M. J. (2021). Artificial intelligence
and machine learning in design of mechanical materials. Mater. Horiz..

[21] Pilania G. (2021). Machine learning
in materials science: From explainable predictions to autonomous design. Comput. Mater. Sci..

[22] Ali Y., Hussain F., Haque M. M. (2024). Advances,
challenges, and future
research needs in machine learning-based crash prediction models:
A systematic review. Accid. Anal. Prev..

[23] Byerly S., Maurer L. R., Mantero A., Naar L., An G., Kaafarani H. M. (2021). Machine learning and artificial intelligence for surgical
decision making. Surg. Infect..

[24] Shwartz-Ziv R., Armon A. (2022). Tabular data: Deep learning is not all you need. Inf. Fusion.

[25] Prokhorenkova, L. ; Gusev, G. ; Vorobev, A. ; Dorogush, A. V. ; Gulin, A. CatBoost: unbiased boosting with categorical features. In Advances in Neural Information Processing Systems, 2018, Vol. 31.

[26] Biau G., Scornet E. (2016). A random forest guided tour. Test.

[27] Chen, T. ; Guestrin, C. Xgboost: A scalable tree boosting system. In Proceedings of the 22nd ACM SIGKDD International Conference on Knowledge Discovery and Data Mining, 2016, pp 785–794.

[28] Antwarg L., Miller R. M., Shapira B., Rokach L. (2021). Explaining anomalies
detected by autoencoders using Shapley Additive Explanations. Expert Syst. Appl..

[29] Zhang C., Bengio S., Hardt M., Recht B., Vinyals O. (2021). Understanding
deep learning (still) requires rethinking generalization. Commun. ACM.

[30] Davis M. E. (2002). Ordered
porous materials for emerging applications. Nature.

[31] Yoon B.-H., Koh Y.-H., Park C.-S., Kim H.-E. (2007). Generation of large
pore channels for bone tissue engineering using camphene-based freeze
casting. J. Am. Ceram. Soc..

[32] Ghosh D., Dhavale N., Banda M., Kang H. (2016). A Comparison of Microstructure
and Uniaxial Compressive Response of Ice-Templated Alumina Scaffolds
Fabricated from Two Different Particle Sizes. Ceram. Int..

[33] Szepes A., Feher A., Szabo-Revesz P., Ulrich J. (2007). Influence of Freezing
Temperature on Product Parameters of Solid Dosage Forms Prepared via
the Freeze-Casting Technique. Chem. Eng. Technol..

[34] Ishizaki, K. ; Komarneni, S. ; Nanko, M. Porous Materials: Process Technology and Applications; Springer Science & Business Media, 2013; Vol. 4.

[35] Szepes A., Fehér A., Szabó-Révész P., Ulrich J. (2007). Influence of Freezing
Temperature on Product Parameters
of Solid Dosage Forms Prepared via the Freeze-Casting Technique. Chem. Eng. Technol..

[36] CatBoost Team Algorithm: Missing Values Processing, CatBoost Documentation. https://catboost.ai/docs/en/concepts/algorithm-missing-values-processing, n.d. (accessed Feb 16, 2025).

[37] Scotti K. L., Dunand D. C. (2018). Freeze casting—A review of processing, microstructure
and properties via the open data repository, FreezeCasting.net. Prog. Mater. Sci..

[38] Lundberg S. M., Lee S. (2017). A unified approach
to interpreting model predictions. arXiv.

